# Current controversies in radiotherapy for breast cancer

**DOI:** 10.1186/s13014-017-0766-3

**Published:** 2017-01-23

**Authors:** David Krug, René Baumann, Wilfried Budach, Jürgen Dunst, Petra Feyer, Rainer Fietkau, Wulf Haase, Wolfgang Harms, Marc D. Piroth, Marie-Luise Sautter-Bihl, Felix Sedlmayer, Rainer Souchon, Frederik Wenz, Rolf Sauer, Breast cancer expert panel of the German Society of Radiation Oncology (DEGRO)

**Affiliations:** 1 Department of Radiation Oncology, University Hospital Heidelberg and National Center for Radiation Research in Oncology (NCRO), Heidelberg, Germany; 2 University Hospital Schleswig-Holstein, Kiel, Germany; 3 Heinrich-Heine-University, Duesseldorf, Germany; 4 Vivantes Hospital Neukoelln, Berlin, Germany; 5 University Hospital Erlangen, Erlangen, Germany; 6 Formerly St.-Vincentius-Hospital, Karlsruhe, Germany; 7 St. Claraspital, Basel, Switzerland; 8 HELIOS-Hospital Wuppertal, Witten/Herdecke University, Wuppertal, Germany; 9 Municipal Hospital, Karlsruhe, Germany; 10 Paracelsus Medical University Hospital, Salzburg, Austria; 11 Formerly University Hospital Tuebingen, Tuebingen, Germany; 12 University Hospital Mannheim, Mannheim, Germany

**Keywords:** Breast cancer, Accelerated partial breast irradiation, Ductal carcinoma in situ, Regional nodal irradiation

## Abstract

Multimodal treatment approaches have substantially improved the outcome of breast cancer patients in the last decades. Radiotherapy is an integral component of multimodal treatment concepts used in curative and palliative intention in numerous clinical situations from precursor lesions such as ductal carcinoma in situ (DCIS) to advanced breast cancer. This review addresses current controversial topics in radiotherapy with special consideration of DCIS, accelerated partial breast irradiation (APBI) and regional nodal irradiation (RNI) and provides an update on the clinical practice guidelines of the Breast Cancer Expert Panel of the German Society of Radiation Oncology (DEGRO).

## Background

Since 2005, the breast cancer expert panel of the German Society for Radiation Oncology (DEGRO) has published and updated evidence-based practical guidelines for the treatment of breast cancer using radiotherapy for the most important clinical situations [1–6].

Interdisciplinary efforts to pursue risk-adapted treatment strategies have improved outcome for breast cancer patients in the last decade. Subsequently, several long-standing paradigms regarding the role of different modalities have been scrutinized.

The most striking example for such a controversy in the radiation oncology community was the introduction of accelerated partial breast irradiation (APBI) for invasive breast cancer, especially intraoperative radiotherapy and the results of the TARGIT A-trial [7, 8]. Another issue of dispute is the role of regional nodal irradiation (RNI) in early stage breast cancer recently highlighted by several randomized studies [9, 10]. Contrary to the results of trials which led to the established role of sentinel lymph-node biopsy, these trials consistently showed a benefit of intensified locoregional treatment, i.e. radiotherapy to the regional lymphatic pathways.

Last but not least, debate continues about the optimal risk-adapted treatment of ductal carcinoma in situ (DCIS). Substantial effort has been dedicated to the establishment of prognostic factors justifying the omission of adjuvant radiotherapy after breast-conserving surgery. Furthermore, some researchers suggest a change in the attitude towards low grade DCIS arguing that it should be regarded as a risk factor for a subsequent breast cancer rather than a malignant disease by itself.

The goal of this article is to provide an update on the DEGRO practical guidelines for APBI, RNI and DCIS with respect to recent data from randomised studies and to discuss their possible impact on future developments.

### Accelerated partial breast irradiation (APBI)

Mostly in order to shorten duration of treatment, APBI is carried out with a variety of techniques for RT delivery. Its feasibility has been proven in randomized controlled trials (RCT) for “anticipated” or postoperative boost radiotherapy in addition to external beam whole breast irradiation (WBI) [11].

Moreover, APBI has been administered as sole RT modality applying different dose and fractionation schemes using various target volume definitions [12]. Summarizing results of these trials, equieffectiveness of the various techniques is unclear and the differences in patient characteristics as well as dosimetric characteristics render the optimal selection of the treatment regimen as well as technique for individual patients difficult.

Table 1 summarizes the results and patient characteristics of the recent randomized controlled trials of ABPI versus WBI followed by a boost to the tumor bed.

**Table 1:** Recent randomized phase III trials studying accelerated partial breast irradiation in breast cancer

	TARGIT A [15, 16]		ELIOT [18]		GEC-ESTRO [14]		Livi et al. [21]	
Technique	kV-IORT (INTRABEAM)		IOERT		Interstitial multicatheter brachytherapy		Intensity-modulated radiotherapy	
Fractionation	40–56 Gy WBI + 10–16 Gy boost	1 × 20 Gy (+ EBRT in 15.2% of patients)	50 Gy WBI + 10 Gy Boost	1 × 21 Gy	50 Gy WBI + 10 Gy Boost	- 8 × 4 Gy in 4 days (HDR) - 7 × 4.3 Gy in 4 days (HDR) - 50 Gy in 3–4 days (PDR)	50 Gy WBI + 10 Gy Boost	6 × 5 Gy in 2 weeks
Inclusion criteria	Unifocal IDC, ≥ 45 years		48–75 years, tumor size ≤ 2.5 cm		DCIS or pT1-2a (<3 cm) pN0/mi, margins ≥ 2 mm (≥5 mm for DCIS and ILC), no lymph-/hemangiosis, ≥ 40 years		Unifocal, ≤ 2.5 cm, > 40 years	
Number of patients	3451		1305		1184		520	
Recruitment period	2000–2012		2000–2007		2004–2009		2005–2013	
Median follow up	2.4 years		5.8 years		6.6 years		5 years	
Local recurrence at 5 years	1.3%	3.3%	0.4%	4.4%	0.9%	1.4%	1.4%	1.5%
	*p* = 0.042 (n.s.) HR n.a.		*p* = 0.0001 HR 9.3 (95% CI 3.3–26.3)		*p* = 0.42 HR n.a.		*p* = 0.86 HR 1.16 (95% CI 0.2–5.8)	
Overall survival at 5 years	94.7%	95.1%	96.8%	96.9%	95.6%	97.3%	96.6%	99.4%
	*p* = 0.099 HR n.a.		*p* = 0.59 HR 1.1 (95% CI 0.7–1.9)		*p* = 0.11 HR n.a.		*p* = 0.057 HR 0.17 (95% CI 0.02–1.36)	

*WBI* whole-breast irradiation, *EBRT* external beam radiotherapy, *HDR* high dose rate, *PDR* pulsed dose rate, *IDC* invasive ductal carcinoma, *ILC* invasive lobular carcinoma, *IORT* intraoperative radiotherapy, *IOERT* intraoperative electron radiotherapy, *DCIS* ductal carcinoma in situ, *HR* hazard ratio, *CI* confidence interval, *n.s*. not significant, *n.a.* not available

#### Multicatheter brachytherapy

Multicatheter brachytherapy is the technique with the longest experience for APBI and the only technique for which 10 year-data from a randomized controlled trial are available [13]. The Budapest trial did show similar results with APBI using multicatheter brachytherapy and WBI with a tumor bed boost, however this mono-institutional trial was closed early due to poor enrollment and the study was hence not adequately powered to show non-inferiority of APBI [13].

Results from the randomized phase III noninferiority-trial on interstitial multicatheter brachytherapy conducted by the GEC-ESTRO group have been published recently [14]. Between 2004 and 2009, 1184 patients were randomized to APBI with with multicatheter brachytherapy or percutaneous WBI of 50 Gy in 2 Gy fractions with a sequential boost of 10 Gy. The primary endpoint was ipsilateral local recurrence. The non-inferiority-margin was set at a difference of 3% at 5 years. At the time of analysis 14 patients had developed a local recurrence at the 5 year-follow up. This corresponded to a 5-year local recurrence rate of 0.9% for EBRT and 1.4% for APBI as sole RT modality (*p* = 0.42). Overall survival was 95.6% for EBRT and 97.3% for APBI (*p* = 0.11). Adverse events occurred less frequently in the APBI-arm, however statistical significance was only reached for breast pain, which occurred in 3.2% after EBRT and 1.1% after APBI (*p* = 0.04). Detailed results on late effects and cosmetic outcome were not yet reported.

#### Intraoperative radiotherapy (IORT)

The TARGIT A-trial tested the approach of intraoperative radiotherapy to the lumpectomy cavity using a 50 kV-device with a spherical applicator [15, 16]. Patients were randomized to WBI with or without a tumor bed boost according to local standards or to IORT with a dose of 20 Gy. However, additional WBI was mandated in the case of additional risk factors identified in the pathologic work-up of the surgical specimen which was the case for about 15% of patients in the IORT-arm. TARGIT A was planned as a non-inferiority-trial with a margin of 2.5% at 5 years. After the first results were published in 2010 with the planned trial cohort size of 2232 patients, recruitment was continued until 2012 with a total size of 3451 patients. The trial protocol was amended in 2004 to allow inclusion of patients in which IORT was administered during a second surgical procedure by reopening the wound (post-pathology-stratum).

The most recent results were published in 2014 with a median follow up of 29 months [15]. 1222 of the patients included in this analysis had a median follow up of 5 years. Results showed an estimated 5-year local recurrence rate of 3.3% with IORT and 1.3% with WBI (*p* = 0.043). This difference was not statistically significant due to adjustment for multiple testing. Furthermore, non-inferiority was established for the whole cohort, but not for the post-pathology stratum which showed a 5-year local recurrence rate of 5.4% with IORT and 1.7% with WBI (*p* = 0.067). The 5-year non-breast cancer-related mortality was higher in the WBI-group (3.5% vs. 1.4%, *p* = 0.089). A recently published report provided further details on subgroup analyses and other endpoints [17]. Tumors without expression of the progesterone receptor had a significantly increased risk of local recurrence in the overall cohort and in the IORT-arm. A multivariate analysis showed that non-inferiority could not be established in the absence of estrogen and/or progesterone receptor expression. The risk of recurrence outside of the index quadrant or the axilla was not elevated in patients in the IORT-arm as compared to patients in the EBRT-arm.

The publication of this trial evoked an unprecedented discussion around the results of this trial in the Lancet and in the Red Journal with criticism circling mainly around the short overall follow up and the statistical design [7].

The second randomized phase III-trial on intraoperative radiotherapy was the ELIOT-study, a single-institutional trial using electrons (IOERT) instead of KV-x-rays [18]. From 2000 to 2007, 1307 patients were enrolled at the European Institute of Oncology in Milan, Italy. The study was designed as an equivalence trial assuming a 5-year local recurrence rate of 3% in the WBI group with an accepted non-inferiority margin of 4.5%. The 5-year ipsilateral breast tumor recurrence rate, although within the prespecified equivalence margin, was significantly higher in the IOERT-arm with 4.4% compared to 0.4% in the WBI-arm (*p* < 0.001). The risk of recurrence was not only elevated in other breast quadrants but also within the index quadrant and regional lymph nodes. There was no difference in distant recurrence or survival. The ELIOT trial provided detailed information about risk factors for local tumor recurrence in the IOERT group. Pathologic tumor size, grading, estrogen receptor status, Ki-67 and molecular subtype were significant predictors of an increased local recurrence rate. In the absence of adverse prognostic factors, the local recurrence rate was 1.1% in patients treated with IOERT. Adverse events occurred less frequently with IOERT with the exception of fat necrosis.

#### External-beam radiotherapy

Interim results from the phase III randomized RAPID-trial suggested that APBI with 3D-conformal EBRT might be associated with inferior cosmesis and an elevated risk of grade 1–2 adverse events [19]. While an interrelation with dosimetric parameters as well as the fractionation scheme of 38.5 Gy in 10 fractions over one week was suspected, a follow up-publication failed to identify treatment-associated predictors of adverse cosmesis in the APBI-group [20].

Livi et al. conducted a randomized phase III-trial using intensity-modulated radiotherapy (IMRT) for APBI at the University of Florence [21]. The trial was designed to detect an 2%-increase in the local recurrence-rate at 5 years from 3% in the standard arm to 5% in the APBI arm. From 2005 to 2013, 520 women were enrolled and randomized to 50 Gy of WBI followed by a boost of 10 Gy or to 30 Gy of APBI in 6 fractions over 2 weeks delivered as IMRT. The clinical target volume for APBI contained the surgical clip as determined by at least 4 clips placed during surgery and an additional margin of 1 cm. The 5 year-local recurrence rate was 1.5% in patients receiving APBI and 1.4% in patients receiving standard treatment (*p* = 0.86). Toxicity and cosmesis were significantly in favor of the APBI-arm.

At the 10^th^ European Breast Cancer Conference held in Amsterdam in April 2016, preliminary results from the IMPORT LOW-trial were presented [22]. This 3-arm randomized phase III trial compared hypofractionated WBI (40.05 Gy in 15 fractions, arm A) to hypofractionated WBI with a lower dose to the breast tissue outside of the tumor bed (40.05 Gy to the tumor bed delivered as a simultaneous integrated boost, 36 Gy to the remaining breast tissue in 15 fractions, arm B) and APBI to the tumor bed (40.05 Gy in 15 fractions, arm C). 2018 patients were recruited. 5-year local recurrence rates were 1.1% for arm A, 0.2% for arm B and 0.5% for arm C. Non-inferiority was demonstrated for the experimental arms B and C (pre-defined threshold 2.5%, one-sided alpha 2.5%).

#### Conclusions of the **DEGRO** expert panel

- APBI is feasible and provides excellent results regarding toxicity and local control in selected low risk-patients.
- Although non-inferiority was established in the TARGIT A- and ELIOT-trial, there are some methodologic issues in the design of those trials including the additional use of EBRT, different timing in the IORT application and the choice of the non-inferiority margin.
- APBI using EBRT should be applied with caution until the results of phase III trials such as NSABP B-39/RTOG 0413, RAPID and IMPORT LOW are published.
- Multicatheter brachytherapy remains the only modality for which 10-year data are available.
- A meta-analysis including 4.489 patients from several of the above mentioned trials demonstrated that overall mortality is not increased with APBI [23].
- Long term follow up is needed to determine the impact of APBI on survival, the risk of late recurrences, adverse events as well as cosmetic results.

### Radiotherapy of the lymphatic pathways in early stage breast cancer

#### Sentinel lymph node metastases

Sentinel lymph node biopsy (SLNB) has replaced axillary lymph node dissection (ALND) as the standard of care for surgical staging of the axilla in patients with clinically unsuspicious lymph nodes [24].

Controversy exists regarding the optimal treatment of alleged low risk patients with 1–2 positive sentinel lymph nodes. The ACOSOG Z0011 trial [25, 26] randomly assigned 891 patients with T1-2 breast cancer with sentinel lymph node metastases after breast-conserving surgery to ALND or no further axillary surgery. All patients were supposed to undergo standard tangential WBI without irradiation of the supra-/infraclavicular region (SCN). The trial was designed as a non-inferiority trial but was closed early due to slow recruitment and low event rates. Overall, 40% patients had micrometastases and 27% of patients in the ALND-arm had additional non-sentinel lymph node metastases. The 10-year results were recently presented at the 2016 ASCO annual meeting and published [27]. Regional nodal recurrence rates were low at 0.5% and 1.5% in the ALND- and SLNB-arms, respectively (*p* = 0.28). There were no significant differences in disease-free or overall survival. However, a retrospective analysis of the available radiation treatment records revealed that there was a significant number of protocol violations regarding radiotherapy [28]. 18.9% of the reviewed patients received radiotherapy to the SCN and more than half of the patients were treated using high tangents known to be associated with a higher radiation dose exposure to axillary lymph node levels I and II compared to conventional tangential irradiation. Protocol violations were equally distributed between the two treatment arms, but it cannot be excluded that RNI contributed to the favorable outcome in the SLNB-arm [29, 30].

The AMAROS-trial [31] conducted by the EORTC addressed a similar patient group as Z0011 (cT1-2 cN0 with sentinel lymph node metastases), however patients with mastectomy were also eligible. 1425 patients were randomly assigned to ALND or radiotherapy to axillary lymph node levels I-III and the SCN. As the event rate was lower than expected, the non-inferiority test for the primary endpoint, the axillary recurrence rate, was underpowered. Yet, axillary recurrence rates at 5 years were low at 0.4% and 1.2% without any differences in disease-free and overall survival. Axillary morbidity was significantly higher in the ALND-arm with lymphedema rates being almost twice as high as in patients treated without ALND.

#### Conclusions of the **DEGRO** expert panel

- There is no clear standard for the treatment of patients with 1–2 sentinel lymph node metastases. It remains uncertain in which patients ALND and RNI including the axilla can safely be omitted.
- Since radiotherapy to the axilla and the SCN yields similar regional recurrence rate and survival compared to axillary dissection while the associated morbidity is reduced, it should always be considered when treatment of the axilla seems indicated.

#### Recent results on radiotherapy of the lymphatic pathways

Several trials on RNI in early stage breast cancer were recently published [32]. Design and results of those trials are summarized in Table 2.

**Table 2:** Recent clinical trials studying regional nodal irradiation in breast cancer

	MA.20 [10]		EORTC 22922/10925 [9]		DBCG-IMN [35]		French trial [36]	
Study design	Phase III randomized controlled trial		Phase III randomized controlled trial		Prospective cohort study		Phase III randomized controlled trial	
Treatment arms	WBI + boost	WBI + boost + SCV/IMN	WBI + Boost TWI	WBI + boost + SCV/IMN TWI + SCV/IMN	WBI + Boost + SCV (left sided tumor location)	WBI + Boost + SCV/IMN (right sided tumor location)	TWI + SCV	TWI + SCV/IMN
Inclusion criteria	N+ or N0 high risk (T3 or T2 with < 10 lymph resected lymph nodes and additional risk factors present)		N+ or N0 (medial or central tumor location)		N+		N+ or N0 (medial or central tumor location)	
Number of patients	1832		4004		3089		1334	
Recruitment period	2000–2007		1996–2004		2003–2007		1991–1997	
Median follow up	9.5 years		10.9 years		8.9 years		8.6 years	
Disease-free survival	77.0%	82.0%	69.1%	72.1%	n.s.		49.9%	53.2%
	*p* = 0.01; HR 0.76 (95% CI 0.61–0.94)		*p* = 0.04; HR 0.89 (95% CI 0.80–1.00)				*p* = 0.35	
Overall survival	81.8%	82.8%	80.7%	82.3%	72.2%	75.9%	59.3%	62.6%
	*p* = 0.38; HR 0.91 (95%-CI 0.72–1.13)		*p* = 0.06; HR 0.87 (95%-CI 0.76–1.00)		*p* = 0.005; HR 0.82 (95%–CI 0.72–0.94)		*p* = 0.8	

*WBI* whole-breast irradiation, *TWI* thoracic wall irradiation, *SCV* supra-/infraclavicular region, *IMN* internal mammary lymph nodes, *HR* hazard ratio, *CI* confidence interval

Generally, two types of trials can be distinguished, those comparing the addition of RNI including the SCN and the internal mammary lymph nodes (IMN) to whole breast or chest wall irradiation to either one alone or those trials addressing treatment of the whole breast or chest wall and the SCN with or without irradiation of the IMN.

The MA.20-[10] and the EORTC 22922/10925-[9] trials belong to the first category. Both trials enrolled mostly patients with 0–3 involved lymph nodes. However, there were several notable differences in the inclusion criteria. While the MA.20-trial only recruited patients after breast-conserving surgery, 24% of patients in the EORTC-trial had undergone mastectomy. Node negative patients were included in both trials, however the EORTC-trial defined medial/central tumor location as a prerequisite for eligibility in these patients whereas the MA.20-trial used a combination of tumor size, number of removed lymph nodes and other risk factors. Hence, the MA.20-trial included only 10% node negative patients while 44% of the study population in the EORTC-trial did not have nodal involvement. Furthermore, there were differences in the radiation technique [3]. Both trials did show significant improvements in locoregional control, distant-metastasis-free survival and disease-free survival. Interestingly, the improvement in distant metastasis-free survival exceeded the improvement in locoregional control, suggesting that RNI could prevent distant spread [3]. Concerning the primary endpoint overall survival, the EORTC-trial was of significance after adjustment for stratification factors (hazard ratio (HR) 0.87, *p* = 0.0496) whereas the MA.20-investigators concluded that there was no significant improvement in overall survival.

While a meta-analysis of both trials [33, 34] showed a significant improvement in overall survival (HR 0.88, *p* = 0.034), subgroup analyses did not yield consistent results on which patient subgroup might benefit most from RNI. In MA.20, the overall survival benefit was most pronounced in estrogen receptor-negative patients (HR 0.69, *p* = 0.05) whereas in the EORTC-trial, this was true for patients who did receive both endocrine and chemotherapy (HR 0.72). These discrepancies might be related to the differences in inclusion criteria regarding type of surgery and node-negative patients (see Table 2).

The DBCG-IMN [35] and the French trial by Hennequin et al. [36] belong to the second category of clinical trials in RNI that specifically addressed the benefit of targeting the IMN-area in RNI. The study of Hennequin et al. [36] is the oldest one of the four mentioned trials. It was designed to detect a 10% benefit in overall survival in the patients treated with RNI including IMN and was thus underpowered to show a difference between the two treatment arms. The DBCG-IMN trial [35] is a prospective cohort study, but was meticiously planned and executed. In patients with right sided breast cancer, the internal mammary region was included into the radiotherapy target volume, whereas in left sided breast cancer patients this region was not treated due to concerns regarding cardiac toxicity. The 8-year overall survival rates were 75.9% with IMN-RT versus 72.2% without IMN-RT (HR 0.82, *p* = 0.005). Subgroup analyses suggested that patients with 1–3 involved lymph nodes in the case of medial/central tumor location and patients with 4 or more involved lymph nodes irrespective of the tumor location did derive the largest benefit [35].

#### Conclusions of the **DEGRO** expert panel

- RNI improves the outcome of patients with early stage breast cancer with locoregional lymph node involvement.
- In patients with 1–3 involved lymph nodes, RNI should be strongly considered, especially in the presence of further risk factors such as negative estrogen or progesterone receptor, poorly differentiated tumors, medial tumor location and premenopausal status.
- In patients with >3 involved lymph nodes, RNI should be regarded as mandatory.
- Irradiation of the IMN should be strongly considered in all patients in whom RNI is performed.
- Currently, due to the differing inclusion criteria for node negative patients in the MA.20- and the EORTC-trials, the optimal selection criteria for RNI in this subgroup remain unclear. However, it can be discussed on a case by case-basis in patients with the above-mentioned risk factors.
- Additional toxicity associated with RNI was mild and none of the mentioned trials showed an increase in cardiac morbidity or mortality, although further follow-up is necessary regarding this endpoint.

### Ductal carcinoma in situ (DCIS)

The effect of adjuvant radiotherapy on local recurrence of DCIS after breast-conserving surgery has been established by 4 randomized controlled phase III trials and a meta-analysis of those trials by the Early Breast Cancer Trialists’ Collaborative Group (EBCTCG) [37].

Several recent publications have shed light on the prognosis and treatment of patients with DCIS. Using data from the Surveillance, Epidemiology and End Results (SEER) registry, Narod et al. investigated the prognosis of more than 108000 women with DCIS diagnosed between 1988 and 2011 [38]. The 20 year-breast cancer-specific mortality was 3.3% overall, but increased to 7.8% for women diagnosed before age 35 years. For women with an ipsilateral invasive recurrence, the relative risk for dying of breast cancer was elevated 18-fold. Radiotherapy did significantly reduce ipsilateral invasive and non-invasive recurrences, but 10-year breast cancer-specific mortality was similar with and without adjuvant radiotherapy.

Another study from the SEER-registry by Sagara et al. used the patient prognostic score developed by Smith et al. [39] to assess the benefit of adjuvant radiotherapy according to different clinical and pathologic factors [40]. Using the patient age (<40 years, 40–60 years, >60 years), tumor size (<16 mm, 16–40 mm, > 40 mm) and grading (low, intermediate, high), more than 32000 patients were assigned a score from 0 to 6. The investigators used propensity score-corrections to account for imbalances in baseline characteristics. Although breast cancer-specific mortality was similar to the analysis by Narod et al. [38], Sagara et al. could show a small but statistically significant benefit in the 10 year breast cancer-specific mortality for patients who received adjuvant radiotherapy. The benefit in terms of breast cancer-specific mortality and overall mortality was highly dependent on the patient prognostic score. Patients with higher scores derived the largest benefit from radiotherapy with absolute improvements in 10 year-breast cancer-specific mortality of 1.9% and 4% in patients with a score of 4 and 5, respectively. While these results suggest that it might be possible to use the patient prognostic score to guide the decision making-process for adjuvant radiotherapy, caution is advised because information on several important factors such as margin status, endocrine therapy and comorbidities were lacking.

Due to the advances in diagnosis and treatment of DCIS as well as declining rates of local recurrence [41], the role of adjuvant radiotherapy has been questioned. Several groups have tried to establish a definition of a low risk-group of patients in which adjuvant radiotherapy after breast-conserving surgery could safely be omitted using clinical, pathological and molecular parameters. However there is no consensus on which factors should be used (for review see [42]).

The ECOG-ACRIN E5194-trial [43] prospectively assessed the outcome of patients with DCIS after breast-conserving surgery without adjuvant radiotherapy. Patients were eligible if they had well or moderately differentiated DCIS ≤ 2.5 cm (cohort 1) or poorly differentiated DCIS ≤ 1 cm (cohort 2) with clear margins (≥3 mm). Although median tumor size was only 6 mm and 7 mm in the two cohorts with wide negative margins for most patients, the 12 year-local recurrence rates were 14.4% and 24.6%, respectively. The 12 year-invasive recurrence rates reached 7.5% and 13.4%, respectively.

The only randomized phase III trial specifically studying the benefit of adjuvant radiotherapy in a prospectively defined „low risk“ population is the RTOG 9804-trial [44]. Women with well or moderately differentiated DCIS with a tumor size of ≤ 2.5 cm were randomly assigned to adjuvant WBI or no radiotherapy after lumpectomy with clear margins. The 7-year local recurrence rate was 0.9% with and 6.7% without adjuvant radiotherapy (*p* < 0.001, HR 0.11). 42% of local failures in the observation arm were invasive recurrences. The rate of contralateral invasive breast cancer (4% at 7 years) was considerably lower than the rate of ipsilateral local failure after radiotherapy, suggesting that radiotherapy could prevent secondary tumors [45]. Toxicity associated with radiotherapy was generally low.

There are several ongoing clinical trials addressing active surveillance [46, 47] or chemoprevention (e.g. CALGB 40903, ClinicalTrials.gov number, NCT01439711) strategies in screening-detected biopsy-proven low or intermediate DCIS. However, this is an experimental approach and should not be chosen outside of clinical trials [48]. A recent retrospective cohort study showed that 20% of patients matching the inclusion criteria for the LORIS-trial [47] after biopsy had invasive carcinoma on final pathology after surgery and 18% of those patients received a recommendation for adjuvant chemotherapy [49].

A systematic review and meta-analysis by Nilsson et al. [50] summarizes the current evidence on hypofractionation and boost radiotherapy for DCIS. Results suggested that moderately hypofractionated radiotherapy (40–42 Gy in 15–16 fractions) is safe with a non-significant trend towards an improvement in local control compared to conventionally fractionated radiotherapy (odds ratio 0.78, *p* = 0.08). There was a benefit of a tumor bed boost in patients with positive margins (odds ratio 0.56, *p* = 0.01). The evidence was mostly limited to small single-institutional retrospective studies with a suboptimal quality of evidence.

A recent multicenter retrospective study presented at the 2016 annual meeting of the American Society for Radiation Oncology included 4131 patients with DCIS [51].

Two thousand six hundred sixty-one patients received a boost to the tumor bed with a median dose of 14 Gy. Local control at 15 years was significantly better in patients receiving a boost (91.6% vs. 88.0%, *p* = 0.013). Patients with positive margins had no significant benefit, however the subgroup was small with limited statistical power.

Two randomized controlled trials, the TROG 07.01 trial (NCT00470236) and the Bonbis trial (NCT00907868), studying the role of a boost irradiation in patients with DCIS have finished patient recruitment. The TROG 07.01 trial additionally addresses hypofractionation compared to standard fractionation in a 2x2 trial design.

A discussion of adjuvant radiotherapy in patients with DCIS should always include consideration of possible late effects of radiotherapy. Cardiotoxicity of adjuvant radiotherapy has been shown to be of relevance especially in patients with left-sided tumor location [52]. There is a clear dose–response relationship [53]. Thus, modern radiotherapy techniques to reduce cardiac radiation doses such as deep inspiration breath hold [54] are of great importance to reduce long term morbidity. Other possible severe late sequelae of radiotherapy include secondary malignancies. Although rare, the risk of secondary malignancies is significantly elevated after radiotherapy for breast cancer. This includes the risk for subsequent lung cancer, esophageal cancer and sarcoma [55]. Smokers should be counseled regarding their elevated risk for both cardiac events and secondary lung cancer after radiotherapy.

#### Conclusions of the **DEGRO** expert panel

- Clinical and pathological factors can help to select patients at lower risk of local recurrence, however there is no uniform definition of a low risk-phenotype.
- Local recurrence rates show no plateau after 10 years of follow up in patients not undergoing radiotherapy.
- Radiotherapy significantly lowers ipsilateral invasive and non-invasive recurrences of DCIS after breast-conserving surgery.
- Patients with high-risk DCIS derive the highest absolute benefit from adjuvant radiotherapy.
- Although both invasive and non-invasive recurrences are significantly reduced by radiotherapy, there is no survival benefit.
- Patients should be informed about the beneficial effects and low toxicity of radiotherapy with optimal local control even in screening-detected DCIS.
- The rate of contralateral invasive breast cancer is considerably lower than the rate of ipsilateral local failure after radiotherapy, suggesting that radiotherapy could prevent secondary tumors.
- The role of boost radiotherapy and hypofractionation is currently unclear, however results from two prospective randomized controlled trials are pending.
- In clinical practice, individual risk factors as well as patient preferences, comorbidities and potential side effects should be carefully weighed against each other in the decision-making process concerning adjuvant radiotherapy.

### Conclusions and future challenges

The treatment of breast cancer is a poster child of how multidisciplinary management can improve the outcome of an oncologic disease. Using data from the National Vital Statistics Reports and the SEER-database, Smith et al. could show that from 1990 to 2007, the breast cancer death rate decreased about 2% per year [56]. The 10 year-breast cancer specific death rate decreased from 29.6% to 20.1% for women treated from 1980–1984 compared to women treated from 1995–1997 (*p* < 0.001) [56].

With improving outcomes, better risk stratification and patient selection for specific treatment approaches, the benefit achieved by individual treatment modalities tends to decrease. Thus, an individual treatment might provide a high relative benefit, but with decreasing absolute benefit and increasing number needed to treat, effects on morbidity and non-breast cancer related mortality gain increasing importance.

The basic goal to optimize treatment outcome may involve different approaches and goals depending on the specific risk-constellation of the disease. While in DCIS and low risk-early stage breast cancer, it may be justified to attempt de-escalation of treatment in order to limit morbidity and preserve quality of life, the contrary i.e. escalation of locoregional (radio-) therapy is indicated in node-positive breast cancer bearing a significant risk of breast cancer-related mortality. Finding the optimal combination of treatment approaches for achieving cure while simultaneously balancing effectiveness against morbidity requires individualization according to the individual risk, comorbidities and personal preferences of the patient. These purposes are most likely achieved by a multidisciplinary approach based on best available evidence.

## Abbreviations

APBI: accelerated partial breast irradiation
CI: confidence interval
DCIS: ductal carcinoma in situ
DEGRO: German Society of Radiation Oncology
EBCTCG: Early Breast Cancer Trialists’ Collaborative Group
EBRT: external beam radiotherapy
ECOG-ACRIN: Eastern Cooperative Oncology Group (ECOG) and American College of Radiology Imaging Network
GEC-ESTRO: Groupe Européen de Curiethérapie and European Society for Radiotherapy & Oncology
HDR: High dose rate
HR: Hazard ratio
IDC: Invasive ductal carcinoma
ILC: Invasive lobular carcinoma
IMN: Internal mammary lymph nodes
IOERT: Intraoperative electron radiotherapy
IORT: Intraoperative radiotherapy
PDR: Pulsed dose rate
RTOG: Radiation Therapy Oncology Group
SCV: Supra-/infraclavicular region
SEER: Surveillance, Epidemiology and End Results
TARGIT: Targeted intraoperative radiotherapy
TROG: Trans Tasman Radiation Oncology Group
TWI: Thoracic wall irradiation
WBI: Whole-breast irradiation
